# Notes and Notices

**Published:** 1891-08

**Authors:** 


### NOTES AND NOTICES.

The Dios Chemical Co., of 914 Locust Street, St. Louis, Mo., have just issued a very handsome colored lithographic plate of the uterus and appendages, showing at a glance the relationships and position of the parts. They will mail it free to any physician upon application.

A New Food.—Lacto-Cereal Food is a new product recently put on the market by Reed & Carnrick, of New York.

It is prepared from milk, cereals, and fruit, and is not only palatable, but highly nutritious and easily digested.

Great progress has been made in recent years in making foods to meet various indications. The Lacto-Cereal Food is especially prepared for invalids, the aged, and for convalescents who need a palatable, digestible, perfect food for building up waste tissues at the least possible expense of digestive effort.—*Dietetic Gazette.*

Dr. Charles F. Stillman and Dr. Arthur B. Hosmer have associated themselves in partnership at 125 State Street, Chicago.

Dr. M. L. Munson has established himself at 1307 Pacific Avenue, Atlantic City, N. J.

"Printers' Ink."—Every issue of this bright little journal is religiously read by many thousand newspaper men and printers, as well as by advertisers. If you want to buy a paper or to get a situation as editor, the thing to do is to announce your desire in a want advertisement. Any story that can be told in twenty-three words can be inserted for two dollars. As a rule, one insertion can be relied upon to do the business. George P. Rowell & Co., Publishers of *Printers' Ink*, 10 Spruce Street, New York.

The New York Medical College and Hospital for Women has just issued its annual announcement for the coming season of 1891 and 1892.

This college presents in combination the following essential elements:

*First.* Unequivocally a *three years' graded course.*

*Second.* Applicants for matriculation must present a Baccalaureate degree from some college or university, or pass an examination in the English branches before the Regents of the University of the State of New York.

*Third.* Students must pass a satisfactory examination at the end of the first year in order to be admitted to the Junior year, and at the end of the Junior year, to be admitted to the Senior year.

*Fourth.* Students are required, before graduation, to pass an examination, not only by the Faculty, but also by a Board of Censors.

Having adopted this high standard, not only as a necessity to the sick, but as a duty to the public, the Trustees and Faculty aim to make this college equal to any in the world. They therefore ask the co-operation of the homœopathic profession, and all friends of the medical education of women, in sending to this college such women as show aptness for a physician's work. It will be their endeavor to give thorough, practical instruction, and they ask attention to the advantages which this institution affords.

For further information address PHŒBE J. B. WAIT, M. D., *Dean and President of the Faculty*, 9th Avenue and 34th Street, New York City.

THE NATIONAL CONSERVATORY OF MUSIC OF AMERICA, Nos. 126 and 128 East 17th Street, New York. The annual entrance examinations of the National Conservatory of Music, Nos. 126 and 128 East 17th Street, New York, will be held as follows: Singing—September 24th and 25th, 1891, from nine A. M. to twelve M.; two to five P. M.; from eight to ten P. M. Violin, 'Cello, Contrabass, Harp, and all other Orchestral Instruments—September 28th, from nine A. M. to twelve M., and two to five P. M. Piano and Organ—September 29th, nine A. M. to twelve M., and two to five P. M. Orchestra—November 2d, from four to six P. M. Chorus—November 4th, from eight to ten P. M. Operatic Chorus—November 2d, from eight to ten P. M. The object of the National Conservatory of Music being the advancement of music in the United States through the development of American talent, applications for admission into the classes of the Conservatory are hereby invited.

CHARLES INSLEE PARDEE, A. M., *Secretary.*

A VICTIM OF ADDISON'S DISEASE.—San Francisco, May 13th. No case in the medical annals of the Coast has excited so much interest as that of George L. Sturtevant, who has just succumbed to Addison's disease, his skin becoming as black as a negro's. His case is the first on record in California, and has novel features. The victim was twenty-one years old and the son of an interpreter at the Merchants' Exchange. Three years ago, when the disease first showed itself, Sturtevant's clear skin was his chief claim to manly beauty. At the time of his death his body was as black as that of a full-blooded negro. The first intimation of the disease was the appearance on the tongue of a black pigment formation of the size of a lead-pencil head. Two months afterward others appeared on the gums, and the skin assumed the saffron hue of jaundice. A diagnosis by experts finally settled the fact that he had Addison's disease.

The father moved to Berkely, where the patient could be secluded and yet have exercise in a large garden. The young man believed he had jaundice, and the fatal nature of his disease was concealed from him. In the second year his skin changed to a bronze tint, and in the third year, from the chest down, he was dead black. He had no pain, and amused himself by reading and playing the piano, but complained of great languor. His case had one peculiarity never before observed. The majority of patients die in the second year, but all who have heretofore passed this stage become insane in the third year. Sturtevant lived the full limit of three years, but showed no signs of insanity. The disease is due to decomposition of the outer coat of the kidneys.

Dr. Skinner takes his holiday this year from August 1st till the 31st October. During August and September his address will be Glencar Hotel, Carah, County Kerry, Ireland, where letters may be sent. During October they should be directed to Waylands, Beckenham, Kent, until further orders. Urgent cases requiring personal attendance had better consult Dr. John H. Clarke, 34 Harrington Road, London, S. W.

25 Somerset Street, London, W. July, 1891.

A Remarkably Successful Operation.—Dr. L. J. Van Marter, of Findlay, O., yesterday operated successfully on both eyes of F. G. Scott, of Delphos, a man ninety-five years of age, who has been blind for twenty years. Sight was restored in both eyes at once. Dr. Van Marter removed both of these cataracts without cutting a piece out of the iris, and, in cutting the capsule, or skin covering the lens, he did so at the periphery or rim of the lens, not at the centre or sight part of it. There is only one other eye surgeon in America who does this operation, and it is regarded as the most difficult thing known to eye surgery. Both the doctor and Mr. Scott are to be congratulated.—*Lima Republican, March 26th.*

Impure Ice.—The danger is that ice contains the same mischievous germs as the water from which it is produced, although in a lesser degree, yet it does contain them. And the opinion entertained that the degree of refrigeration necessary to produce congelation would cause the death of micro-organisms, was an erroneous opinion.

Not to accumulate figures and details of little interest to the reader, I limit myself to the results of experiments on the bacillus of typhoid fever. A temperature of 0° C. has only a very limited action on the microbe, as will be seen. Eleven days after congelation, the cold having been constantly and rapidly maintained and the ice not allowed to liquefy, one cubic centimeter, which has contained innumerable bacilli, artificially multiplied, still contains more than a million. After twenty-seven days three hundred and thirty-six thousand (I take round numbers); after forty-two days, ninety thousand; after seventy-seven days, seventy-two thousand; after one hundred and three days, seven thousand.

Consequently, water containing this typhus bacillus remains impure, and contains this bacillus alive when taken as ice. Experimentally this bacillus is only destroyed, rendered inactive, by subjecting the liquid to alterations of congealing and melting.

Artificial ice, which is recommended hygienically as being superior to natural ice, will not actually possess this quality unless it has been manufactured from water that was perfect in all respects. A. Cartaz, M. D.

The Mattison Prize.—With the object of advancing scientific study and settling a now mooted question, Dr. J. B. Mattison, of Brooklyn, offers a prize of $400 for the best paper on "Opium Addiction as Related to Renal Disease," based upon these queries: Will the habitual use of opium in any form produce organic renal disease? If so, what lesion is most likely? What is the rationale? The contest is to be open for two years from December 1st, 1890, to either sex, and any school or language. The prize paper is to belong to the American Association for the Cure of Inebriety, and be published in a New

York medical journal, *Brooklyn Medical Journal*, and *Journal of Inebriety*. Other papers presented are to be published in some leading medical journal, as their authors may select. All papers are to be in possession of the Chairman of Award Committee, on or before January 1st, 1893. The Committee of Award will consist of Dr. Alfred L. Loomis, President of N. Y. Academy of Medicine, Chairman; Drs. H. F. Formad, Philadelphia; Ezra H. Wilson, Brooklyn; George F. Shrady, and Joseph H. Raymond, editor *Brooklyn Medical Journal*.

THE HOMŒOPATHIC MEDICAL SOCIETY, OF THE STATE OF OREGON, held their fifteenth annual meeting in parlors G and H of Hotel Portland, May 13th and 14th. There was a full attendance of physicians from all parts of the State.

The following officers were elected: B. E. Miller, M. D., President; Osman Royal, M. D., First Vice-President; H. C. Jefferds, M. D., Second Vice-President; Orpha D. Baldwin, M. D., Recording Secretary; H. F. Stevens, M. D., Corresponding Secretary; C. L. Nichols, M. D., Treasurer; Drs. H. B. Drake, C. E. Geiger, George Wigg, C. A. Macrum, and S. A. Brown, Board of Censors.

Drs. C. H. Day, P. L. Mackenzie, and J. J. McMicken were elected members of the Society.

The afternoon session of the first day was opened by an address of welcome delivered by Dr. C. A. Macrum. This was followed by the annual address of the President, Dr. George Wigg, who has so ably and faithfully served the Society for several years. In a pleasant and forcible manner he reminded the members of their duty as guardians of the public health, and the necessity for constant and untiring efforts in their search for means of alleviating the suffering and restoring the sick.

A Committee was appointed by the President for the purpose of endeavoring to influence the Legislature for a separate State Licensing Board, or proper representation on the one already existing. H. F. STEVENS, *Sec'y.*

